# What future research should bring to help resolving the debate about the efficacy of EEG-neurofeedback in children with ADHD

**DOI:** 10.3389/fnhum.2014.00321

**Published:** 2014-05-15

**Authors:** Madelon A. Vollebregt, Martine van Dongen-Boomsma, Dorine Slaats-Willemse, Jan K. Buitelaar

**Affiliations:** ^1^Karakter University Centre for Child and Adolescent PsychiatryNijmegen, Netherlands; ^2^Department of Cognitive Neuroscience, Donders Institute for Brain, Cognition and Behaviour, Radboud University Medical CentreNijmegen, Netherlands; ^3^Centre for Cognitive Neuroimaging, Donders Institute for Brain, Cognition and Behaviour, Radboud University Medical CentreNijmegen, Netherlands; ^4^Department of Psychiatry, Donders Institute for Brain, Cognition and Behaviour, Radboud University Medical CentreNijmegen, Netherlands

**Keywords:** EEG-neurofeedback (EEG-NF), attention-deficit/hyperactivity disorder (ADHD), efficacy, methodology, non-pharmacological interventions

## Abstract

In recent years a rising amount of randomized controlled trials, reviews, and meta-analyses relating to the efficacy of electroencephalographic-neurofeedback (EEG-NF) in children with attention-deficit/hyperactivity disorder (ADHD) have been published. Although clinical reports and open treatment studies suggest EEG-NF to be effective, double blind placebo-controlled studies as well as a rigorous meta-analysis failed to find support for the efficacy of EEG-NF. Since absence of evidence does not equate with evidence of absence, we will outline how future research might overcome the present methodological limitations. To provide conclusive evidence for the presence or absence of the efficacy of EEG-NF in the treatment of ADHD, there is a need to set up a well-designed study that ensures optimal implementation and embedding of the training, and possibly incorporates different forms of neurofeedback.

Attention-deficit/hyperactivity disorder (ADHD) is the most common neurodevelopmental disorder, affecting about 5% of all children worldwide. ADHD is characterized by a pattern of inattention and/or hyperactivity and impulsivity (Polanczyk et al., 2007).

While medication is the most effective treatment in ADHD (Faraone and Buitelaar, 2010), it also entails a number of concerns. Firstly, side effects have been reported and for some serious and life-threatening side effects, the risk is not clear and will likely remain so due to the rarity of these events (Graham et al., 2011). Children with ADHD and their parents also have significant reservations about possible negative long-term effects of medication (Berger et al., 2008). Secondly, there is insufficient evidence of long-term efficacy of medication for ADHD (van de Loo-Neus et al., 2011). Thirdly, the symptoms of ADHD have been found to reappear after discontinuing drug treatment (Jensen et al., 2007; Murray et al., 2008). These misgivings about ADHD medications have contributed to the interest in developing non-pharmacological approaches to treatment, such as electroencephalographic-neurofeedback (EEG-NF). EEG-NF is based on the rationales that (1) the neural basis of ADHD is characterized by deviant EEG patterns that play a role in the pathophysiology of the disorder; and (2) voluntary modulation of specific brain activity patterns can be learned by operant learning strategies. The first rationale originates from the finding that the majority of resting state electroencephalography (EEG) in children with ADHD is characterized by increased slow-wave activity (primarily in theta range) and decreased fast-wave activity (primarily in beta range). These slow and fast waves are often coupled, resulting in elevated theta/alpha and theta/beta ratios (see Barry et al., 2003 for a review). Although a deviant theta/beta ratio has been found in ADHD rather consistently (Arns et al., 2013), the exact role of such a deviant pattern in the pathophysiology of ADHD is not clear yet (van Dongen-Boomsma, 2014). In addition, although voluntary modulation of specific brain activity patterns might be possible, clear-cut proof is still lacking. If these rationales are correct, they will provide for a rational neuroscience-based treatment of ADHD that brings about normalization of the underlying neural abnormality and thereby clinical improvement.

## EEG-NF; the current state of affairs

In recent years, a number of randomized controlled trials, reviews, and meta-analyses relating to EEG-NF in children with ADHD have been published. Although particularly non-blinded studies conclude that EEG-NF is probably effective, robust evidence based on methodologically sound studies is still lacking. The majority of studies did not include a placebo group and/or blinded measures. Studies that did include a placebo group or a blinded design have not found superior effects of EEG-NF compared to placebo-NF (Perreau-Linck et al., 2010; Arnold et al., 2012; van Dongen-Boomsma et al., 2013,^1^). In addition, a systematic review and meta-analysis of randomized controlled trials (RCTs) of non-pharmacological interventions in children with ADHD including EEG-NF studies, reported non-significant results for the blind rating of symptoms (ES 0.29, *p* = 0.07; CI = −0.02, 0.61) (Sonuga-Barke et al., 2013). One of our previous studies (Vollebregt et al., 2013) did not find any effects at a neurocognitive level following EEG-NF treatment of ADHD participants. This paper also included a systematic review of the extant literature which indicated that our findings were in line previous studies.

However, absence of evidence does not equate with evidence of absence. If EEG-NF truly has no effect, then the possibility of regulating brain activity via EEG-NF to improve ADHD symptoms can be refuted. Alternatively, a true effect of EEG-NF may be hidden by methodological flaws which would imply that the optimal way to apply or study this therapy is not yet known.

Improvements in a number of different areas will be needed to overcome discrepancies in the EEG-NF literature. Firstly, improvements will be needed in study-design. Secondly, the implementation and embedding of the training may have to be improved. Thirdly, the assessment of other forms of neurofeedback, alongside EEG will also help to clarify outstanding questions. These three levels of recommendations will be discussed below.

## Study-design

While placebo-controlled RCTs are the gold standard in pharmacological research, there is no consensus regarding the optimal design for EEG-NF experiments.

### Placebo-controlled RCT’s

A major advantage of the inclusion of a placebo condition is that all aspects of both treatments are identical except for the underlying hypothesized working element. This enables allocation of positive findings to the working element only. Another advantage is that the amount of expectancy is equal between groups in contrast to all other control condition options, in which an equal amount of expectancy is difficult or even impossible to assess and correct for. The inclusion of a placebo condition also allows blindness of the child and parents, making blind assessments by proximal individuals possible. A common misconception of placebo-controlled RCTs which also exists in EEG-NF research (e.g., Heinrich et al., 2007; Gevensleben et al., 2012) is that it would be unethical to deprive participants of an effective treatment by allocating them to the placebo condition instead of the treatment condition. However, in cases where the efficacy of a treatment is not known and the purpose of the study is to determine if the treatment may be effective, then allocation of participants to a placebo group does not involve depriving a participant of treatment, as long as medication which a participant may be taking for his or her condition is continued during the course of the experiment.

A randomized placebo-controlled trial also has drawbacks, namely the fact that it is time- and energy- intensive, expensive, and may not be the strongest design for all interventions or settings (West and Spring, 2014).

Applying a randomized placebo-controlled trial design to EEG-NF experiments might create a selection bias. In certain cases, placebo-controlled RCT’s may thereby limit the external validity of the findings (West et al., 2008). Only people that are willing to accept that they may be allocated to the placebo group will participate in the study. However, this problem may partly be alleviated by ensuring that participants that are taking medication continue their regime unaltered throughout the duration of the study. In addition, including a placebo condition may make it more difficult to recruit participants due to a potential participant’s reluctance to receive the placebo treatment. This can be (partially) overcome by conducting a multi-site center study and allowing ADHD medication to be used through the study period. Furthermore, lowering the expectancy by the possibility of allocation to the placebo group may make it more difficult for the treatment to have a positive effect of the treatment (like neuroregulation) (Gevensleben et al., 2009). In accordance, most participants of EEG-NF placebo-controlled RCTs conducted until now seem to experience the treatment as a placebo condition (Logemann et al., 2010; Lansbergen et al., 2011; van Dongen-Boomsma et al., 2013; Vollebregt et al., 2013). One might speculate that this absence of efficacy is caused by reduced motivation of the participants or—on the other hand—from flaws in the protocol. The feasibility of the training should therefore be rated by evaluating EEG indices during the sessions of both groups (i.e., learning curves), in addition to measuring the guessing rate (i.e., how well parent and child were able to guess to what group they were allocated), as well as analyzing the differences between pre and post quantitative EEG measurements. Until now, of the randomized placebo-controlled trials, Vollebregt et al. was the only study that evaluated EEG indices during the sessions and did not show any learning effect.

Generally, a placebo condition is only justified if the condition meets the following criteria. Firstly, the placebo condition must be inert with no possibility that this treatment trains a measurable physiological effect. This should be assessed by analyzing EEG indices during the sessions in the placebo condition. Secondly, all participants (i.e., the child, his/her parents, teacher(s)) as well as all examiners (i.e., the raters, but also the EEG-NF therapist) should be blinded. Due to technical restrictions in placebo-controlled studies it has not been possible to blind the therapist while implementing manual thresholding. However, the promising proposal by Kerson (2013) has overcome these restrictions by creating a design in which real-time noise is superimposed on the placebo data creating the illusion of real time EEG recordings.

### Alternatives to placebo-controlled RCT’s

In relation to psychotherapy research, problems with the use of placebo-conditions have been emphasized (Borkovec and Sibrave, 2005). Clinical trials which attempt to eliminate unspecific treatment components by the use of placebo-conditions, might give an inaccurate estimate of the clinical value of the treatment if nonspecific variables (e.g., expectations) interact with active treatment components. Jeopardizing treatment fidelity in such a way might also happen in EEG-NF. All problems discussed in relation to psychotherapy can certainly not readily be generalized to the research of EEG-NF in which the target of training is non-psychological in nature, in contrast to the psychological target psychotherapy has. Nevertheless, internal validity should not be readily assumed in either design. The external validity of alternatives to placebo-controlled RCT is often stronger than of placebo-controlled RCTs, but they also face a serious limitation in terms of ensuring internal validity (West et al., 2008). A number of promising alternatives to placebo-controlled RCTs, which attempt to overcome these difficulties, do exist. Disadvantages of performing a placebo-controlled RCT in certain situations and possible solutions to deal with them were elaborately discussed by West and Spring (2014). Their points and arguments on alternatives to placebo-controlled RCT’s will be used further to discuss the use of such alternatives to study EEG-NF. When studying EEG-NF, the placebo condition can for instance be replaced with “additive comparison” or “treatment dismantling” in which aspects that are hypothesized to contribute to the efficacy of the treatment are added or left out of the treatment respectively. Alternatives to random assignment could be time-series, counterbalanced, cross-over and group randomized designs. These alternatives avoid unfair allocation and thereby circumvent a selection bias. “Partial blinding” is a method which allows for manual thresholding while minimizing the number of people that have to be unblinded. Another option is an “equipoise design” in which two treatments are equally well valued at the onset of the study which makes blinding less relevant.

A concrete example of an alternative approach to a placebo-controlled RCT for EEG-NF is “interrupted time series analysis” in which the treatment is introduced at different time points, but endpoints are equal (West et al., 2008). If the rater is unaware of the duration of treatment, the measurement can still be blind and expectancies of parents and children are controlled relatively well, i.e., they all receive the treatment that they expect to be effective. This design allows blind measures and comparable expectations in each group despite the lack of a placebo group. However, the design does assume that the amount of time spent on the training predicts the amount of improvement.

### The optimal design

Regardless of the manner in which internal validity is maximized, the study design can also be improved in other areas. For instance, the sample size should be in congruence with the power analysis, thereby enhancing the power and allowing more analyses (such as subtype analyses). In addition, the study-design should seek to determine whether or not EEG-NF is efficacious as a monotherapy or alternatively is valuable as an add-on therapy received in conjunction with medication. Although few studies to date have compared medication to neurofeedback (Duric et al., 2012; Meisel et al., 2013; Ogrim and Hestad, 2013), these studies struggled with major limitations and inconsistent findings. A more thorough comparison between medication and EEG-NF can be achieved by including additional subgroups that assess participants without medication together with participants on medication. A strong design should furthermore obtain objective measures of ADHD symptoms, e.g., by using school observations by an independent observer, actometers, or neurocognitive tests. Finally, a strong design should have an optimal implementation and embedding of the treatment, discussed further below.

In summary, an improved design can be achieved by addressing the above mentioned points either through a placebo controlled RCT or an alternative design. Importantly, a design can only be optimal if reliable and valid outcome measures are selected and good quality control is maintained throughout data collection. Internal validity should be maximized while bias should be minimized (West and Spring, 2014).

## Implementation and embedding of the training

### EEG deviation

Most EEG-NF protocols focus on ADHD-related deviation in frequency bands during rest; up-regulation of theta power and down-regulation of beta power (Monastra et al., 2005). While the majority of children with ADHD exhibit diminished beta-power, a subgroup of children with ADHD have been found to have excessive beta-power (Arns, 2012). Thus, the idea of repairing a deviate EEG pattern would not apply on these children without a personalized protocol.

### Reward feedback

The percentage positive feedback that should be given has been under debate. Some researchers argue that for instance 80% positive feedback would to be too high for optimal learning (Arns et al., 2014). The percentage should not be too high not allowing sufficient learning, neither should the percentage be too low preventing a feeling of control. Consensus on what this percentage should be has not been reached and should be investigated.

### Learning paradigms

To further improve the training, the development of a paradigm with instructions that are clearly goal-directed and in which the participant is encouraged to actively attempt to reach a certain “brain-state” might be more effective than strictly following an operant learning principle in which learning occurs through performance rather than through following a preceding intention. Creating awareness of the desired behavior might not only be more effective during training itself, but might also facilitate transfer into daily life since the participant is actively aware how to achieve a goal. Achievement of explicit goals might in addition enhance motivation. Since no placebo-controlled studies until now have been able to show specific treatment effects, a possible explanation besides design-related explanations discussed above, could be that a paradigm lacking clear instructions might not lead to a learned behavior being able to be incorporated in a participant’s daily life.

### Transfer

Without transfer of the (during treatment) learned skills into daily life, the usefulness of EEG-NF can seriously be questioned. To facilitate potential transfer effects into daily life, the following recommendations can be made. Explicit feedback on the deviation of oscillations might enable awareness of how to minimize this deviation, thereby creating a possibility to consciously prompt this minimization in any situation in daily life as well. This could be further strengthened by implementing transfer trials; a block within the training in which no immediate feedback is given. The participant is required to act as if immediate feedback is given at that moment, even though feedback is only given after the block has ended. In this way a daily life situation, in which no immediate feedback is provided either, is simulated more realistically. The implementation of transfer trials has already been applied (e.g., Strehl et al., 2006; Drechsler et al., 2007; Heinrich et al., 2007; Leins et al., 2007; Gevensleben et al., 2009, 2014), but has not been studied in a sufficiently well designed trial. Finally, transfer effects can be optimized by combining the pure EEG-NF sessions with sessions including behavioral therapeutic aspects to teach the participant to recognize daily life situations in which to apply the new skills learned from the EEG-NF (Heinrich et al., 2007; Gevensleben et al., 2009). Despite different aspects of EEG-NF that have been under debate as discussed above, a clear consensus of how the optimal implementation of EEG-NF should look like has not been reached.

## Different forms of neurofeedback

Of course, the conventional EEG-NF is not the only alternative treatment for ADHD that could be studied. Different methods than the most popular most practiced resting state oscillatory EEG-NF could be scrutinized. Examples are online tomographic NF (tNF) computed from multichannel scalp EEG (Liechti et al., 2012), real-time functional magnetic resonance imaging neurofeedback (fMRI-NF) (Sulzer et al., 2013) or magnetoencephalographic neurofeedback (MEG-NF) (Foldes et al., 2011). The advantage of tNF is that more specific brain regions can be targeted due to the use of more electrodes. At least the same advantage can be reached when using MEG, without all the preparatory hustle that usually comes along with EEG. fMRI is of course spatially even more precise but deals with a temporal delay of measurement. Both MEG and fMRI based neurofeedback are far more expensive than EEG; however difference in costs may be less when only a few sessions are needed. Studies have shown that all these methods are feasible, each having its own advantages and disadvantages. All these methods seem to outperform conventional EEG-NF since they allow more direct feedback, based on more specific brain structures.

When sticking to the EEG-NF protocol or more specifically to a personalized EEG-NF protocol, it can be questioned how deviations should be determined. Children in the active group of our study received a personalized protocol, but EEG data recorded during the sessions showed that not all desired training directions were met (Vollebregt et al., 2013). Significant improvement on group level can only solidly be interpreted if all training conditions hypothesized to improve ADHD (either on behavioral or neurocognitive level) are actually improved in the desired direction. Determining deviations during rest might differ from deviations during task performance. Generalization to daily life might be greater when the neurofeedback is based on EEG deviations during task performance. The most often replicated EEG-deviation in ADHD has been shown at rest (Arns et al., 2013), but does not show an unambiguous relationship with behavioral and cognitive performance (van Dongen-Boomsma, 2014). Still, the existence of a straightforward relationship between these two is the basis of the conventional EEG-NF therapy. Since dysfunction due to the core ADHD-symptoms is primarly experienced during cognitive or motor activity, a focus on electrophysiological indices during activity may have a better rationale than during rest. In addition, generalization to daily life (hence, transfer) might be greater when the neurofeedback is based on EEG deviations during task performance. These arguments plead for real-time deviation determined during interactive task performance. A clear example of such an application of neurofeedback (in healthy individuals) is by real-time training alpha oscillations during task performance in an MEG scanner (e.g., Jensen et al., 2011).

In the early days, alpha enhancement neurofeedback (6–13 Hz) protocols failed to find a specific effect on hyperkinetic behavior (Nall, 1973). After this starting point, the alpha frequency band has not been the focus of neurofeedback. Nevertheless, alpha activity is associated with active inhibition of brain areas, which is hypothesized to result in allocation of attention (Klimesch et al., 2007). Aberrant modulation of alpha activity during task performance has been associated with attention problems on clinical level (i.e., adults with ADHD) (ter Huurne et al., 2013). Hence, a relationship has actually been shown between behavioral measures (to what extent the cue induced allocation of attention) and alpha oscillations (the lateralized difference in alpha power expected due to allocation of attention following the inhibition notion) in ADHD. In addition, the height of the alpha frequency peak has been shown to be lower in a subgroup of children with ADHD (Vollebregt et al., in press) and predictive to treatment outcome of several treatments (Ulrich et al., 1984; Arns et al., 2008, 2009, 2012; Arns, 2012). Different characteristics of the alpha frequency band therefore seem to be relevant to ADHD. It is worthwhile to further investigate neurofeedback possibilities training this frequency band. These results could be related to the neurophysiological substrate of the disorder. To study this active inhibition notion, active task involvement is necessary implying interactive task performance. By improving the therapy with suggestions mentioned above, other forms of neurofeedback might also have potential as treatment for ADHD.

## Conclusion

The debate whether EEG-NF is an effective treatment for ADHD can be closed by setting up an optimal study with a study-design that tackles the drawbacks of a randomized placebo-controlled trial design that are consequential to studying EEG-NF while keeping blind measurements and avoiding other ways of desecrating the internal validity. In addition, EEG-NF should be implemented in an optimal learning setting both on the technical level of the EEG-NF and with respect to embedding of the learning strategies into daily life. Finally, alternative forms of neurofeedback to conventional EEG-NF, may offer other, maybe even better, promising alternatives.

## Conflict of interest statement

In the past 3 years, Jan K Buitelaar has been a consultant to/member of advisory board of/and/or speaker for Janssen Cilag BV, Eli Lilly, Bristol-Myer Squibb, Shering Plough, UCB, Shire, Novartis, and Servier. He is neither an employee nor a stock shareholder of any of these companies. He has no other financial or material support (e.g., expert testimony, patents or royalties). The authors have been supported by BrainGain, a Dutch research consortium, funded by Smartmix, an initiative of Netherlands Organization for Scientific Research (NWO) to examine the effectiveness of EEG-neurofeedback in children with ADHD.

## Footnotes

^1^The study by van Dongen-Boomsma et al. (2013) describes behavioral data acquired from a project registered in the Clinical trial register under “Project ADHD and EEG-Neurofeedback THERapy” www.clinicaltrials.gov; NCT00723684. Lansbergen et al. (2011) describe the pilot data of this project. Vollebregt et al. (2013) describe the neurocognitive data of this project and in addition provide a systematic review.
